# Effect of WW Domain-Containing Oxidoreductase Gene Polymorphism on Clinicopathological Characteristics of Patients with EGFR Mutant Lung Adenocarcinoma in Taiwan

**DOI:** 10.3390/ijerph182413136

**Published:** 2021-12-13

**Authors:** Ju-Pi Li, Jinghua Tsai Chang, Po-Chung Ju, Ming-Hong Hsieh, Yu-Hua Chao, Thomas Chang-Yao Tsao, Ming-Ju Hsieh, Shun-Fa Yang

**Affiliations:** 1 School of Medicine, Chung Shan Medical University, Taichung 402, Taiwan; jupili@csmu.edu.tw (J.-P.L.); cshy841@csh.org.tw (P.-C.J.); mhhpsy@hotmail.com (M.-H.H.); nka6150@gmail.com (Y.-H.C.); his885889@gmail.com (T.C.-Y.T.); 2 Department of Pediatrics, Chung Shan Medical University Hospital, Taichung 402, Taiwan; 3 Institute of Medicine, Chung Shan Medical University, Taichung 402, Taiwan; jinghuat@csmu.edu.tw; 4 Department of Medical Research, Chung Shan Medical University Hospital, Taichung 402, Taiwan; 5 Department of Psychiatry, Chung Shan Medical University Hospital, Taichung 402, Taiwan; 6 Division of Chest, Department of Internal Medicine, Chung Shan Medical University Hospital, Taichung 402, Taiwan; 7 Oral Cancer Research Center, Changhua Christian Hospital, Changhua 500, Taiwan; 8 Graduate Institute of Biomedical Sciences, China Medical University, Taichung 404, Taiwan; 9 College of Medicine, National Chung Hsing University, Taichung 402, Taiwan

**Keywords:** lung cancer, *WWOX*, polymorphism, EGFR mutation

## Abstract

Lung adenocarcinoma is the most common histological type of non-small cell lung cancer, which accounts for the majority of lung cancers. Previous studies have showed that dysregulation of WW domain-containing oxidoreductase (*WWOX*) participates in the generation of several cancer types, including lung cancer. However, whether these *WWOX* polymorphisms are related to the clinical risk of epidermal growth factor receptor (EGFR)-mutated lung adenocarcinoma is worthy of investigation. The present study examined the relationship between the *WWOX* single-nucleotide polymorphisms (SNPs; rs11545028, rs12918952, rs3764340, rs73569323, and rs383362) and the clinicopathological factors in lung adenocarcinoma patients with or without EGFR mutations. We found that there was no significant difference in the genotype distribution of *WWOX* polymorphism between EGFR wild-type and EGFR mutant in patients with lung adenocarcinoma. Our results demonstrated that the presence of at least one G genotype (CG and GG) allele on *WWOX* rs3764340 was associated with a significantly higher risk of nearby lymph node involvement in those patients harboring EGFR mutations (odds ratio (OR) = 3.881, *p* = 0.010) compared with the CC genotype. Furthermore, in the subgroup of lung adenocarcinoma patients with the EGFR-L858R mutation, both *WWOX* rs3764340 C/G (OR = 5.209, *p* = 0.023) and rs73569323 C/T polymorphisms (OR = 3.886, *p* = 0.039) exhibited significant associations with the size of primary tumors and the invasion of adjacent tissues. In conclusion, these data indicate that *WWOX* SNPs may help predict tumor growth and invasion in patients with EGFR mutant lung adenocarcinoma, especially those with the EGFR-L858R mutant in Taiwan.

## 1. Introduction

Lung cancer has a higher incidence and mortality rate among cancers in the world [1]. There are two main types of lung cancer: non-small cell lung cancer (NSCLC) and small cell lung cancer. NSCLCs account for approximately 80% to 85% of lung cancers, and adenocarcinoma is the most common histological type of NSCLC. Many risk factors are currently known to cause lung cancer, among which mutations in the epidermal growth factor receptor (EGFR) gene have been shown to be related to more than 60% of NSCLCs [2]. EGFR tyrosine kinase inhibitors (TKIs) have been introduced into the first-line treatment of NSCLCs to improve treatment efficacy and overall survival [3]. The short in-frame deletions of exon 19 (Exon19 in-frame deletion) mutation and the single-point substitution of leucine by arginine at codon 858 (L858R) mutation are located in the tyrosine kinase domain and account for approximately 90% of all EGFR mutations in lung adenocarcinoma [4,5]. Although EGFR kinase mutations are associated with increased sensitivity to TKIs, not all cancers with these mutations are associated with increased therapeutic outcomes. Therefore, it is important to further analyze whether additional genetic variants are synergistically involved in these lung adenocarcinoma patients with EGFR mutations.

The WW domain-containing oxidoreductase (*WWOX)* gene is mapped to human chromosome 16q23.3–24.1, which spans approximately 1.1 megabases and consists of nine exons and eight introns [6,7]. The *WWOX* gene encompasses the FRA16D fragile region, which frequently undergoes chromosomal breaks and rearrangements in cancers [8,9,10]. The *WWOX* gene mainly encodes a protein of 414 amino acids (46 kDa). Its N-terminal region contains two tandem WW domains and its C-terminal region contains a catalytic domain homologous to short-chain dehydrogenase/reductase family proteins [11]. The *WWOX* protein has been shown to participate in a variety of cellular processes, such as DNA damage responses, cellular metabolism, and tumor suppression [7,12,13,14]. Over time, numerous studies support that *WWOX* exerts tumor suppressor effects through specific molecular actions that are mostly cell type specific [15]. Dysregulation of *WWOX* not only leads to tumorigenesis and cancer progression, but also causes genome instability and treatment difficulties [16]. The reduction or absence of *WWOX* expression has been found to be associated with several cancers, such as breast cancers, thyroid cancer, oral cancer, lung cancer, and so on [8,17,18,19,20,21,22].

Furthermore, it is known that genetic variants, such as single nucleotide polymorphisms (SNPs), are highly correlated with cancer susceptibility [23]. Several *WWOX* SNPs, including rs11545028, rs12918952, rs3764340, and rs383362, have been demonstrated to act as a potential risk factor for various malignant cancers [20,24,25,26]. In contrast, *WWOX* rs73569323 SNP is negatively associated with the risk of hepatocellular carcinoma [26]. Therefore, whether these *WWOX* polymorphisms are related to the clinical risk of EGFR-mutated lung adenocarcinoma is worthy of investigation. In the present study, we evaluated the effects of *WWOX* polymorphisms, including rs11545028, rs12918952, rs3764340, rs73569323 and rs383362, on lung adenocarcinoma with wild-type and mutant EGFR. In addition, we assessed the associations between *WWOX* SNPs and the clinicopathological factors in lung adenocarcinoma patients with or without EGFR mutations.

## 2. Materials and Methods

### 2.1. Patients and Specimens

A total of 316 patients with lung adenocarcinoma were recruited at Chung Shan Medical University Hospital, Taiwan. All patient data, including gender, age and exposure to exogenous risk factors, such as smoking status, were obtained from medical records and questionnaires. Genomic DNA was collected from tumor specimens or whole blood specimens for EGFR gene sequencing or *WWOX* genotyping, respectively. Lung adenocarcinoma is staged at the time of diagnosis according to the tumor/lymph node/metastasis (TNM) classification of malignant tumors of the American Joint Committee on Cancer. The size of the primary tumor and the invasion of adjacent tissues are described as T, T1-T4; the regional lymph nodes’ status is described as N; and distant metastasis is described as M [27]. The study protocol was approved by the Institutional Review Board of Chung Shan Medical University Hospital (No. CS1-20144, 24 August 2020). Each participating patient provided a signed informed consent form before the start of the study.

### 2.2. EGFR Gene Sequencing

Genomic DNA was extracted from the tumor tissues of patients with lung adenocarcinoma using the QIAamp Fast DNA Tissue Kit (Qiagen, Valencia, CA, USA) according to the manufacturer’s instructions. Exons 18–21 of the *EGFR* gene were amplified using the polymerase chain reaction (PCR) and were then subjected to the DNA sequencing reaction using the ABI PRISM 3130XL System (Applied Biosystems, Foster City, CA, USA) as described previously [28].

### 2.3. Genotyping of WWOX Polymorphisms

Genomic DNA was collected from whole-blood specimens of patients with lung adenocarcinoma using the QIAamp DNA Blood Mini Kit (Qiagen, Valencia, CA, USA) according to the manufacturer’s protocols. The allelic identification of five *WWOX* polymorphisms (rs11545028, rs12918952, rs3764340, rs73569323 and rs383362) was examined using the TaqMan SNP genotyping assay and the ABI StepOnePlus Real-Time PCR system (Applied Biosystems, Foster City, CA, USA). For a negative control, each PCR reaction used distilled water instead of DNA in the reaction system.

### 2.4. Statistical Analysis

The Mann–Whitney U test and Fisher’s exact test were used to compare the differences in clinical characteristics and genotype distribution frequencies between lung adenocarcinoma patients harboring the EGFR wild-type or mutation type in this study. Multiple logistic regression models after controlling for other variables were used to estimate the odds ratios (ORs) with corresponding 95% confidence intervals (CIs) of the association between the genotype frequencies and risk of EGFR types. All data were analyzed using SAS software, version 9.1 (SAS Institute Inc., Cary, NC, USA). A *p*-value less than 0.05 was used to indicate statistical significance.

## 3. Results

### 3.1. Characteristics of Study Participants

To explore the effects of *WWOX* polymorphisms on lung adenocarcinoma risk, a total of 316 lung adenocarcinoma patients were enrolled. These lung adenocarcinoma patients were divided into patients with EGFR wild type (*n* = 127, 40.2%) and patients with EGFR mutation (*n* = 189, 59.8%) based on EGFR sequencing data. Table 1 showed the clinical characteristics of these enrolled patients. The significant differences in gender (*p* < 0.001) and cigarette smoking status (*p* < 0.001) were observed between the patients with lung adenocarcinoma with the EGFR wild type and those with the EGFR mutation (Table 1). Compared with the EGFR wild type group, there were more female patients (*n* = 120, 63.5%) and never smokers (*n* = 145, 76.7%) in the EGFR mutant group (Table 1). The significance was not observed between groups in the other clinical characteristics, such as age, tumor stage, tumor T status, lymph node status, and distant metastasis (Table 1).

### 3.2. Distribution Frequency of WWOX Genotypes in Lung Adenocarcinoma Patients

Whole-blood specimens from the recruited 316 patients were collected and examined for the five *WWOX* genotype distribution frequencies (rs11545028, rs12918952, rs3764340, rs73569323 and rs383362). The genotype distributions and associations of *WWOX* gene between the lung adenocarcinoma patients with the EGFR wild type and those with the EGFR mutation were shown in Table 2. In these lung adenocarcinoma patients, whether it was EGFR wild type or mutation, the alleles with the highest distribution frequency at rs11545028, rs12918952, rs3764340, rs73569323 and rs383362 were homozygous for CC, homozygous for GG, homozygous for CC, homozygous for CC, and homozygous for GG, respectively. In order to minimize the interference of confounding variables, a multiple logistic regression model was used to estimate the adjusted odds ratio (AOR) with a 95% confidence interval (CI) after adjusting for variances such as age and smoking habits. Table 2 showed that there was no significant difference in the genotype distribution of *WWOX* polymorphisms between EGFR wild type and EGFR mutant in lung adenocarcinoma patients.

We further studied the genotype distributions and associations of the *WWOX* gene in lung adenocarcinoma patients with two EGFR hotspot mutations, L858R (*n* = 89) and exon19 in-frame deletion (*n* = 91). In comparison with the EGFR wild type group, no significant association was found between *WWOX* polymorphisms and the two EGFR hotspot mutations in lung adenocarcinoma patients (Table 3).

### 3.3. Correlation between WWOX SNPs and the Tumor Classification in Lung Adenocarcinoma Patients

Lung adenocarcinoma is staged according to the tumor/lymph node/metastasis (TNM) classification of the American Joint Committee on Cancer, an internationally recognized system [27]. Next, we studied the association between the frequency of *WWOX* genotype distribution and clinicopathological characteristics in the recruited patients. As shown in Table 4, it was found that *WWOX* rs3764340 was positively correlated with lymph node status in the EGFR mutation group. Compared with the CC genotype, the presence of at least one G genotype (CG and GG) allele on *WWOX* rs3764340 was associated with a significantly higher risk of nearby lymph node involvement in the patients harboring EGFR mutations (*n* = 189, OR: 3.881, 95% CI: 1.295–11.629, *p* = 0.010; Table 4).

We further performed a subgroup analysis based on the two EGFR hotspot mutations: L858R and Exon19 in-frame deletion. In the L858R subgroup, patients with at least one G genotype (CG and GG) of *WWOX* rs3764340 had the higher risk of nearby lymph nodes (*n* = 89, OR = 5.209, 95% CI = 1.111–24.424, *p* = 0.023; Table 4). Compared with the CC genotype, at least G genotype (CG and GG) allele on *WWOX* rs3764340 also enhanced the risk of the tumor status, including the size of the primary tumor and the invasion of adjacent tissues in patients with EGFR L858R mutation (OR = 3.681, 95% CI = 1.259–10.757, *p* = 0.014; Table 4). Furthermore, we observed that rs73569323 carrying at least T genotype (CT and TT) allele was significant associated with tumor status of lung adenocarcinoma patients with EGFR L858R mutation (OR = 3.886, 95% CI = 1.000–15.103, *p* = 0.039; Table 5). The data demonstrated that lung adenocarcinoma patients with EGFR L858R mutation, accompanied by *WWOX* rs73569323 C > T polymorphism, may show large tumor size and invasion of adjacent tissues.

## 4. Discussion

In the present study, we found that the frequency of *WWOX* SNPs rs11545028, rs12918952, rs3764340, rs73569323, and rs383362 was not significantly different between wild-type and mutant EGFR lung adenocarcinoma patients. The existence of at least one G genotype (CG and GG) allele on *WWOX* rs3764340 exhibited a significant association with the involvement of nearby lymph nodes in the lung adenocarcinoma patients harboring *EGFR* mutations, particularly those with the *EGFR* L858R mutation. In the lung adenocarcinoma patients with the *EGFR* L858R mutation, the *WWOX* rs3764340 G genotype or rs73569323 T genotype of *WWOX* was shown to be associated with a significantly increase of primary tumor size and invasion of adjacent tissues. Our data suggest-that examining *WWOX* SNPs, rs3764340 and rs73569323, may help identify patients with lung adenocarcinoma with EGFR L858R mutations that have a higher risk of tumor size (T) and affected lymph node (N) status.

*WWOX* has been proven to perform tumor suppression through several molecular mechanisms in different cancers and diseases. The germline inactivation of the *WWOX* gene through homologous deletion can lead to human autosomal recessive diseases, such as the *WWOX*-related epileptic encephalopathy syndrome [15]. Iliopoulos et al. [29] show that changes in *WWOX* expression in lung, breast, and bladder cancers are not only due to genomic alterations, such as loss of heterozygosity and homozygous deletions, but also epigenetic modifications, such as promoter hypermethylation. *WWOX* allele deletions have been found in 30.0% (three out of ten) lung adenocarcinomas [17]. Indeed, the incidence of *WWOX* exon 6–8 deletion is reported to be high in Chinese NSCLC patients [30]. Furthermore, hypermethylation in the promoter region of *WWOX* gene and mutation of this gene may be related to NSCLC carcinogenesis [31]. Donati et al. [19] shows that *WWOX* protein is lost or reduced in most NSCLCs and loss of *WWOX* expression is associated with to highly aggressive tumors using immunohistochemistry analysis. Multiple pathogenic copy number variations spanning of *WWOX* have been identified associated with neurological conditions [32]. Here, we found that *WWOX* SNPs exhibited significant associations with the size of the primary tumor and the invasion of adjacent tissues in patients with EGFR mutant lung adenocarcinoma, especially those with EGFR-L858R mutant.

Lung adenocarcinoma has been shown to be related to many risk factors, such as genetic and environmental factors. Previous studies have shown that EGFR gene mutations occur more frequently in Asians than in Caucasians [33]. Furthermore, the significant differences in gender and smoking status of the lung adenocarcinoma patients recruited in this study were similar to previous studies. That is, lung adenocarcinoma has been found to occur more frequently in non-smokers and Asian women [34,35]. The genotype distribution of *WWOX* polymorphisms showed no significant difference between wild type and mutant EGFR in lung adenocarcinoma patients. The FRA16D fragile region, which contains the *WWOX* gene, is a hot spot for genomic instability, prone to chromosomal breakage and copy number variations [9,10]. The use of sequencing analysis has found high levels of SNPs in the *WWOX* gene and several missense polymorphisms in cancer cell lines and tumor tissues [36]. Here we showed that two *WWOX* SNPs, rs3764340 C/G and rs73569323 C/T, are highly associated with large tumor size and invasion of adjacent tissues in patients with EGFR-L858R mutant lung adenocarcinoma. However, we did not observe the association between the other *WWOX* SNPs and the risk of lung adenocarcinoma, which may be attributed to the limited samples.

We found that the lung adenocarcinoma patients with both EGFR mutation and *WWOX* rs3764340 C/G polymorphism may be prone to the involvement of nearby lymph nodes, especially those with EGFR L858R mutation. In patients with EGFR-L858R mutant lung adenocarcinoma, *WWOX* rs3764340 C/G polymorphism is also associated with tumor growth and invasion. Previous studies have shown that the C/G genotype of *WWOX* rs3764340 is highly associated with differentiated thyroid carcinoma [20], elevated grade and metastasis risk of osteosarcoma [25], and is susceptible to cervical invasive cancer [37]. The rs3764340 C/G polymorphism causes the amino acid at codon 282 of *WWOX* to replace proline to alanine. We speculate that the variant allele 282 may affect the biological functions of *WWOX*, thereby making individuals susceptible to cancer.

Our data demonstrated that *WWOX* rs73569323 polymorphism is positively associated with tumor size and invasion in patients with EGFR-L858R mutant lung adenocarcinoma. In contrast, *WWOX* rs73569323 SNP is found to decrease the risk of hepatocellular carcinoma [26]. The rs73569323 SNP is located in the 3’ untranslated region (UTR), which is usually one of the microRNA (miRNA) target regions. MiRNAs that negatively regulate the expression of target genes have an important regulatory role in carcinogenesis [38]. In head and neck squamous cell carcinoma (HNSCC), miR-134 expression was reversely associated with the *WWOX* expression in clinical HNSCC tissues and miR-134 directly targets the 3′ UTR of *WWOX* gene in HNSCC cells [39]. Interestingly, Chen et al. [40] show that the transfection of miR-134 mimics decreased the level of *WWOX*, whereas, anti-miR-134 increased *WWOX* expression in the small cell lung cancer cells. In addition, miR-24 also directly binds to the 3’ UTR of *WWOX* gene to inhibit gene expression in NSCLC cells [41]. Therefore, *WWOX* rs73569323 SNP may affect the various miRNA binding to 3′ UTR, leading to different expression and function of *WWOX* in different cancer types.

There are some limitations in the present study. Further studies on larger cohorts are needed to confirm the association between these *WWOX* SNPs and the clinicopathological characteristics of patients with lung adenocarcinoma. Since our study only analyzes the Taiwanese patient group, it may be necessary to conduct larger population-based studies in different ethnic groups for verification in the future. Moreover, it is necessary to study whether these polymorphic changes alter the function of *WWOX* protein in EGFR-mutated lung adenocarcinoma patients, especially L858R-mutated lung adenocarcinoma patients.

## 5. Conclusions

The present results indicate that *WWOX* rs3764340 SNP is associated with a significantly increased risk of lymph node involvement in patients with lung adenocarcinoma carrying EGFR mutations. In addition, *WWOX* rs3764340 and rs73569323 polymorphisms are highly correlated with the size of the primary tumor and the invasion of adjacent tissues in Taiwanese lung adenocarcinoma patients carrying the EGFR-L858R mutation gene. Our data suggest that *WWOX* SNPs may help to predict tumor growth and invasion in patients with EGFR mutant lung adenocarcinoma, especially those with the EGFR-L858R mutant. In Taiwanese patients with EGFR-mutant lung adenocarcinoma, especially those with EGFR-L858R gene mutation, the underlying mechanism of the *WWOX* signaling cascade dysregulation is worthy of further exploration.

## Figures and Tables

**Table 1:** Demographics and clinical characteristics of 316 patients in lung adenocarcinoma with EGFR mutation status.

Variable	Wild Type (*N* = 127) *n* (%)	EGFR Mutation (*N* = 189) *n* (%)	*p* Value
**Age**			
Mean + SD	65.23 + 13.29	66.32 + 13.44	*p* = 0.479
**Gender**			
Male	79 (62.6%)	69 (36.5%)	*p* < 0.001
Female	48 (37.8%)	120 (63.5%)	
**Cigarette smoking status**			
Never-smoker	55 (43.3%)	145 (76.7%)	*p* < 0.001
Ever-smoker	72 (56.7%)	44 (23.3%)	
**stage**			
I + II	29 (22.8%)	50 (26.5%)	*p* = 0.466
III + IV	98 (77.2%)	139 (73.5%)	
**Tumor T status**			
T1 + T2	65 (51.2%)	115 (60.8%)	*p* = 0.089
T3 + T4	62 (48.8%)	74 (39.2%)	
**Lymph node status**			
Negative	32 (25.2%)	60 (31.7%)	*p* = 0.209
Positive	95 (74.8%)	129 (68.3%)	
**Distant Metastasis**			
Negative	59 (46.5%)	85 (45.0%)	*p* = 0.795
Positive	68 (53.5%)	104 (55.0%)	

**Table 2:** Distribution frequency of *WWOX* genotypes of 127 EGFR wild type and 189 EGFR mutation type in lung adenocarcinoma patients.

Variable	Wild Type (*N* = 127) (%)	Mutation Type (*N* = 189) (%)	OR (95% CI)	AOR (95% CI)
**rs11545028**				
CC	72 (56.7%)	96 (50.8%)	1.00	1.00
CT	44 (34.6%)	83 (43.9%)	1.415 (0.879–2.278)	1.610 (0.860–3.013)
TT	11 (8.7%)	10 (5.3%)	0.682 (0.275–1.693)	0.757 (0.222–2.578)
CT + TT	55 (43.3%)	93 (49.2%)	1.268 (0.807–1.993)	1.446 (0.796–2.626)
**rs12918952**				
GG	117 (92.1%)	172 (91.0%)	1.00	1.00
GA	10 (7.9%)	14 (7.4%)	0.952 (0.409–2.217)	1.019 (0.352–2.945)
AA	0 (0%)	3 (1.6%)	---	---
GA + AA	10 (7.9%)	17 (9.0%)	1.156 (0.512–2.614)	1.229 (0.443–3.410)
**rs3764340**				
CC	107 (84.3%)	157 (83.1%)	1.00	1.00
CG	18 (14.2%)	30 (15.9%)	1.136 (0.603–2.141)	1.315 (0.585–2.955)
GG	2 (1.5%)	2 (1.0%)	0.682 (0.095–4.913)	1.195 (0.099–14.461)
CG + GG	20 (15.7%)	32 (16.9%)	0.966 (0.763–1.225)	1.304 (0.599–2.836)
**rs73569323**				
CC	105 (82.7%)	166 (87.8%)	1.00	1.00
CT	22 (17.3%)	23 (12.2%)	0.661 (0.351–1.246)	0.622 (0.258–1.500)
TT	0 (0%)	0 (0%)	----	----
CT + TT	22 (17.3%)	23 (12.2%)	0.661 (0.351–1.246)	0.622 (0.258–1.500)
**rs383362**				
GG	97 (76.4%)	155 (82.0%)	1.00	1.00
GT	26 (20.5%)	32 (16.9%)	0.770 (0.433–1.371)	0.626 (0.292–1.341)
TT	4 (3.1%)	2 (1.1%)	0.313 (0.056–1.741)	0.207 (0.023–1.884)
GT + TT	30 (23.6%)	34 (18.0%)	0.709 (0.408–1.233)	0.563 (0.271–1.170)

The AORs with 95% CIs were estimated by multiple logistic regression models after controlling for age and smoking.

**Table 3:** The associations between the polymorphisms of *WWOX* and the EGFR hotspot mutations in lung adenocarcinoma patients.

Variable	Wild Type		L858R	Exon 19 in-Frame Deletion	
	(*N* = 127) (%)	(*N* = 89) (%)	OR (95% CI)	(*N* = 91) (%)	OR (95% CI)
**rs11545028**					
CC	72 (56.7%)	42 (47.2%)	1.00	51 (56.0%)	1.00
CT	44 (34.6%)	44 (49.4%)	1.714 (0.874–3.017)	33 (36.3%)	1.059 (0.595–1.885)
TT	11 (8.7%)	3 (3.4%)	0.263 (0.468–1.772)	7 (7.7%)	0.898 (0.326–2.475)
CT + TT	55 (43.3%)	47 (52.8%)	1.465 (0.850–2.525)	40 (44.0%)	1.027 (0.597–1.767)
**rs12918952**					
GG	117 (92.1%)	80 (89.9%)	1.00	83 (91.2%)	1.00
GA	10 (7.9%)	8 (9.0%)	1.170 (0.443–3.093)	6 (6.6%)	0.846 (0.296–2.418)
AA	0 (0%)	1 (1.1%)	---	2 (2.2%)	---
GA + AA	10 (7.9%)	9 (10.1%)	1.316 (0.512–3.384)	8 (8.8%)	1.128 (0.427–2.979)
**rs3764340**					
CC	107 (84.3%)	71 (79.8%)	1.00	78 (85.7%)	1.00
CG	18 (14.2%)	16 (18.0%)	1.340 (0.641–2.800)	13 (14.3%)	0.991 (0.458–2.141)
GG	2 (1.5%)	2 (2.2%)	1.507 (0.207–10.946)	0 (0%)	---
CG + GG	20 (15.7%)	18 (20.2%)	1.356 (0.671–2.742)	13 (14.3%)	0.892 (0.418–1.901)
**rs73569323**					
CC	105 (82.7%)	79 (88.8%)	1.00	78 (85.7%)	1.00
CT	22 (17.3%)	10 (11.2%)	0.604 (0.271–1.348)	13 (14.3%)	0.795 (0.377–1.677)
TT	0 (0%)	0 (0%)	----	0 (0%)	----
CT + TT	22 (17.3%)	10 (11.2%)	0.604 (0.271–1.348)	13 (14.3%)	0.795 (0.377–1.677)
**rs383362**					
GG	97 (76.4%)	74 (83.1%)	1.00	72 (79.1%)	1.00
GT	26 (20.5%)	13 (14.6%)	0.655 (0.315–1.362)	19 (20.9%)	0.985 (0.506–1.915)
TT	4 (3.1%)	2 (2.3%)	0.655 (0.117–3.675)	0 (0%)	---
GT + TT	30 (23.6%)	15 (16.9%)	0.655 (0.329–1.306)	19 (20.9%)	0.853 (0.445–1.635)

**Table 4:** Associations between polymorphic genotypes of *WWOX* (rs3764340) and clinicopathologic characteristics of lung cancer with EGFR mutation.

Variable	EGFR Mutation (*N* = 189)			L858R (*N* = 89)			Exon 19 in-Frame Deletion (*N* = 91)		
	CC (*N* = 157)	CG + GG (*N* = 32)	*p* Value	CC (*N* = 71)	CG + GG (*N* = 18)	*p* Value	CC (*N* = 78)	CG + GG (*N* = 13)	*p* Value
**Stage**									
I + II	43 (27.4%)	7 (21.9%)	*p* = 0.519	22 (31.0%)	4 (22.2%)	*p* = 0.465	20 (25.6%)	3 (23.1%)	*p* = 0.844
III + IV	114 (72.6%)	25 (78.1%)		49 (69.0%)	14 (77.8%)		58 (74.4%)	10 (76.9%)	
**Tumor T status**									
T1 + T2	99 (63.1%)	16 (50.0%)	*p* = 0.168	53 (74.6%)	8 (44.4%)	*p* = 0.014 *^,b^	42 (53.8%)	8 (61.5%)	*p* = 0.606
T3 + T4	58 (36.9%)	16 (50.0%)		18 (25.4%)	10 (55.6%)		36 (46.2%)	5 (38.5%)	
**Lymph node status**									
Negative	56 (35.7%)	4 (12.5%)	*p* = 0.010 *^,a^	28 (39.4%)	2 (11.1%)	*p* = 0.023 *^,c^	27 (34.6%)	2 (15.4%)	*p* = 0.168
Positive	101 (64.3%)	28 (87.5%)		43 (60.6%)	16 (88.9%)		51 (65.4%)	11 (84.6%)	
**Distant Metastasis**									
Negative	71 (45.2%)	14 (43.8%)	*p* = 0.879	39 (45.2%)	8 (43.8%)	*p* = 0.426	29 (37.2%)	6 (46.2%)	*p* = 0.538
Positive	86 (54.8%)	18 (56.2%)		32 (54.8%)	10 (56.2%)		49 (62.8%)	7 (53.8%)	

* *p* value < 0.05 as statistically significant. ^a^ OR (95% CI): 3.881 (1.295–11.629); ^b^ OR (95% CI): 3.681 (1.259–10.757); ^c^ OR (95% CI): 5.209 (1.111–24.424).

**Table 5:** Associations between polymorphic genotypes of *WWOX* (rs73569323) and clinicopathologic characteristics of lung cancer with EGFR mutation.

Variable	EGFR Mutation (*N* = 189)			L858R (*N* = 89)			Exon 19 in-Frame Deletion (*N* = 91)		
	CC (*N* = 166)	CT + TT (*N* = 23)	*p* Value	CC (*N* = 79)	CT + TT (*N* = 10)	*p* Value	CC (*N* = 78)	CT + TT (*N* = 13)	*p* Value
**Stage**									
I + II	46 (27.7%)	4 (17.4%)	*p* = 0.293	23 (29.1%)	3 (30.0%)	*p* = 0.954	2 (28.2%)	1 (7.7%)	*p* = 0.115
III + IV	120 (72.3%)	19 (82.6%)		56 (70.9%)	7 (70.0%)		56 (71.8%)	12 (92.3%)	
**Tumor T status**									
T1 + T2	105 (63.3%)	10 (43.5%)	*p* = 0.069	57 (72.2%)	4 (40.0%)	*p* = 0.039 *^,a^	44 (56.4%)	6 (46.2%)	*p* = 0.491
T3 + T4	61 (36.7%)	13 (56.5%)		22 (27.8%)	6 (60.0%)		34 (43.6%)	7 (53.8%)	
**Lymph node status**									
Negative	54 (32.5%)	6 (26.1%)	*p* = 0.534	27 (34.2%)	3 (30.0%)	*p* = 0.792	26 (33.3%)	3 (23.1%)	*p* = 0.462
Positive	112 (67.5%)	17 (73.9%)		52 (65.8%)	7 (70.0%)		52 (66.7%)	10 (76.9%)	
**Distant Metastasis**									
Negative	78 (47.0%)	7 (30.4%)	*p* = 0.135	43 (54.4%)	4 (40.0%)	*p* = 0.389	32 (41.0%)	3 (23.1%)	*p* = 0.218
Positive	7 (53.0%)	16 (69.6%)		36 (45.6%)	6 (60.0%)		46 (59.0%)	10 (76.9%)	

* *p* value < 0.05 as statistically significant. ^a^ OR (95% CI): 3.886 (1.000–15.103).

## Data Availability

The datasets generated for this study are available on request to the corresponding authors.
